# A perforator-based adipocutaneous skin paddle derived from free muscle flap reconstruction as a hypothesis-generating human tissue model of early localized lymphatic insufficiency

**DOI:** 10.1038/s41598-026-64775-3

**Published:** 2026-08-05

**Authors:** Katja K. Koll, Martin M. Zimmermann, Matthias Schulte, Adriana C. Panayi, Gabriel Hundeshagen, Ulrich Kneser, Christoph Hirche

**Affiliations:** 1 Department of Hand, Plastic and Reconstructive Surgery, Microsurgery, Burn Centre BG Klinik Ludwigshafen, Ludwigshafen, Germany; 2 Medical Faculty of the University Heidelberg, Heidelberg, Germany; 3 Department of Cranio-Maxillofacial and Oral Surgery, University Hospital Zurich, University of Zurich, Zürich, Switzerland; 4 Department of Plastic Surgery, Hand, and Reconstructive Microsurgery, BG Unfallklinik Frankfurt am Main gGmbH, Affiliated Hospital of Goethe-University, Frankfurt am Main, Germany

**Keywords:** Secondary lymphedema, Early lymphatic insufficiency, Human lymphedema model, Lymphangiogenesis, Inflammation, Fibrosis, Free muscle flaps, Diseases, Immunology, Medical research

## Abstract

Secondary lymphedema remains poorly understood, particularly with regard to early human tissue responses after lymphatic outflow disruption. This study evaluates the perforator-based adipocutaneous skin paddle (PBASP) from vascularized free muscle flaps as a hypothesis-generating human tissue platform for early localized lymphatic insufficiency. The PBASP, receives arterial and venous perfusion but is not reconnected to physiological lymphatic outflow and was therefore hypothesized to develop changes consistent with early local lymphatic outflow insufficiency. Tissue samples were collected from 15 patients who underwent free muscle flap transfer, with PBASP samples obtained 7–14 days post-surgery. Histological, immunofluorescence, molecular, and protein analyses were performed to assess lymphangiogenesis, inflammation, extracellular matrix remodeling, and adipogenesis. PBASP tissue exhibited epidermal thickening, dermal expansion, subcutaneous matrix loosening, and leukocyte infiltration. Immunofluorescence showed a 3.6-fold increase in LYVE-1 + lymphatic vessel cross-sectional area. Gene expression analysis revealed upregulation of markers like VEGF-C, VEGFR3, IL-6, and MMP-9. ELISA demonstrated increased LYVE-1, IL-6, and COL1A1 protein levels. Together, PBASP tissues showed structural, lymphatic-marker, inflammatory, and early remodeling changes consistent with early localized lymphatic outflow insufficiency. Together, these findings support the PBASP-derived approach as a paired human tissue platform for studying early, localized lymphatic insufficiency and for generating mechanistic hypotheses relevant to secondary lymphedema.

**Supplementary Information:**

The online version contains supplementary material available at 10.1038/s41598-026-64775-3.

## Introduction

Secondary lymphedema (sLE), a common complication of oncologic treatments, arises from lymphatic tissue damage or lymph node dissection, affecting 20–40% of patients undergoing oncologic surgery with lymphadenectomy. While not life-threatening, lymphedema is a progressive condition that significantly impacts quality of life, function, body image, and can lead to lifelong stigma^1^.

Iatrogenic-induced lymphatic vessel damage leads to lymphatic stasis, driven by upregulated VEGF-C, which promotes lymphangiogenesis by increasing the expression of lymphangiogenesis markers like PROX1 and LYVE1^2^. Chronic stasis causes progressive tissue changes, including thickening of the epidermis, dermis, and subcutaneous layers, along with dilation of lymphatic vessels^3–5^.

Inflammatory cytokines—like TGF-β, IFN-γ, IL-6, TNF-α, and IL-1—further drive disease progression^6,7^, contributing to extracellular matrix (ECM) remodeling through increased collagen deposition and MMP-9 expression, ultimately leading to fibrosis and lymphatic dysfunction^8,9^. Lymphatic stasis also promotes adipogenesis, marked by adipocyte hypertrophy and increased adiponectin expression^10^.

Despite advances in understanding its mechanisms, sLE remains poorly understood at the molecular level, and current treatments are primarily symptomatic or surgical^11–14^. Rodent models offer limited translatability, underscoring the need for human-relevant models^5,15^.

**Fig. 1:**
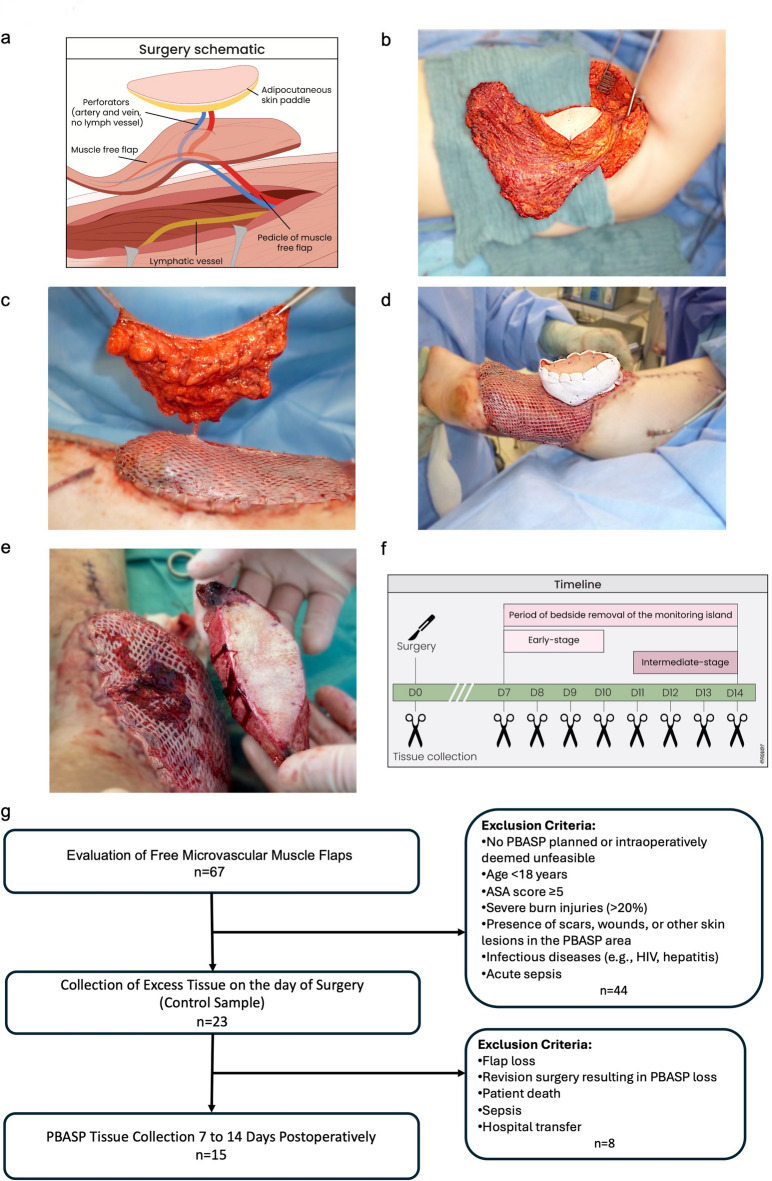
Schematic and intraoperative images of a free muscle flap with perforator-based adipocutaneous skin paddle (PBASP). (**a**) Schematic illustration of a free muscle flap with a perforator-based adipocutaneous skin paddle (PBASP), showing its vascular supply and the intended absence of physiological lymphatic outflow after transfer. (**b**–**e**) Intraoperative images of a free muscle flap with PBASP. (**b**) The latissimus dorsi flap is elevated, with the PBASP included to monitor blood supply. (**c**) The PBASP is connected to the muscle flap solely through arterial and venous blood vessels. (**d**) The muscle flap reconstructs a lower limb tissue defect, with the PBASP temporarily secured to monitor blood supply for 7–14 days. The exposed tissue margins of the PBASP are covered with a synthetic skin replacement dressing (EpiGARD) to prevent desiccation. (**e**) Collection of PBASP Tissue through bedside ligation and removal. (**f**) Schematic timeline of the study, indicating tissue collection at the time of surgery and at the time of PBASP removal. PBASP tissue collection was performed once per patient at the time of bedside removal of the monitoring island, within the predefined postoperative window of Days 7–14. The timeline illustrates the possible sampling window and does not indicate daily serial sampling from the same patient. (**g**) Flow diagram illustrating the sample collection process, including criteria for exclusion.

In plastic surgery, soft tissue defects are often reconstructed using free muscle flaps, a technique that transplants muscle tissue from healthy donor sites to the affected area^16^. Perfusion monitoring of these tissue transplants can be achieved using a perforator-based adipocutaneous skin paddle (PBASP) (Fig. 1a). Designed as a skin island based on a single perforator, the PBASP is identified preoperatively via duplex ultrasonography and confirmed intraoperatively (Fig. 1b,c). It is fix onto the muscle flap temporarily during flap transfer (Fig. 1d), allowing bedside visual monitoring. Ligation and removal are typically performed bedside after 7–14 days (Fig. 1e,f)^17,18^. At the BG Trauma Center Ludwigshafen, this method has been standard practice for over 10 years, offering a simple and effective way to assess flap viability without added surgical burden.

Although the PBASP receives arterial and venous blood supply^19^, it lacks lymphatic drainage, potentially resulting in lymphedema formation (Fig. 1a). This clinical application suggests that PBASP may provide a promising hypothesis-generating in-human tissue platform for studying early responses to presumed local lymphatic outflow insufficiency, while not representing an established equivalent of chronic human lymphedematous tissue.

## Results

### Clinical specimens and patient characteristics

Over a 12-month period, 67 free muscle flap surgeries with prospectively scheduled PBASP analysis were performed. In 15 cases, both initial and follow-up PBASP samples were harvested between postoperative day 7 and 14 (Fig. 1g). The cohort included 10 males and five females, with a mean age of 63.5 years (SD 19.0) and a mean BMI of 30.4 (SD 5.0). The Latissimus Dorsi flap was the most commonly utilized free muscle flap. For additional patient and procedural characteristics, see Table 1. Relevant patient-level comorbidities and clinical characteristics with potential influence on inflammatory and lymphangiogenic responses are summarized in Supplementary Table 2. PBASP tissues collected between Days 7 and 14 were classified as interventional samples, while Day 0 samples, representing intraindividual skin without intervention of respective patients, served as controls. To assess temporal changes, follow-up samples were categorized as early-stage (Days 7–10) or intermediate-stage (Days 11–14) (Fig. 1f). Unless otherwise specified, ‘PBASP’ refers to the pooled intervention cohort comprising all PBASP samples harvested between postoperative Days 7 and 14.

**Table 1:** Demographic and clinical characteristics of study participants.

Age, y, mean (SD)	63.5 (19.0)
Sex, no. (%)	
Male	10 (66.7)
Female	5 (33.3)
Flap type, no. (%)	
Latissimus dorsi muscle flap	11 (73.33)
Gracilis flap	2 (13.33)
Rectus muscle flap	2 (13.33)
BMI, kg/m^2^, mean (SD)	30.4 (5.0)
Days of PBASP removal, mean, (SD)	10 (2.4)
Days of PBASP removal < 11 days, no. (%)	9 (60.0)
Days of PBASP removal > 11 days, no. (%)	6 (40.0)
Mean flap ischemia time, min (SD), *n* = 7	69.3 (17.5)

Summary of demographic and clinical characteristics of patients included in the study. Mean age is presented with standard deviation (SD). Flap types included Latissimus Dorsi, Gracilis, and Rectus muscle flaps with adjacent, perforator-based adipocutaneous paddles. Body Mass Index (BMI) and days to PBASP removal are presented as mean values with SD. Percentages for sex distribution, flap types, timing of PBASP removal and flap ischemia time are also provided.

y, years; BMI, Body mass index; PBASP, perforator-based adipocutaneous skin paddle; min, minutes.

### Swelling, epidermal and dermal alterations indicate structural changes in PBASP

**Fig. 2:**
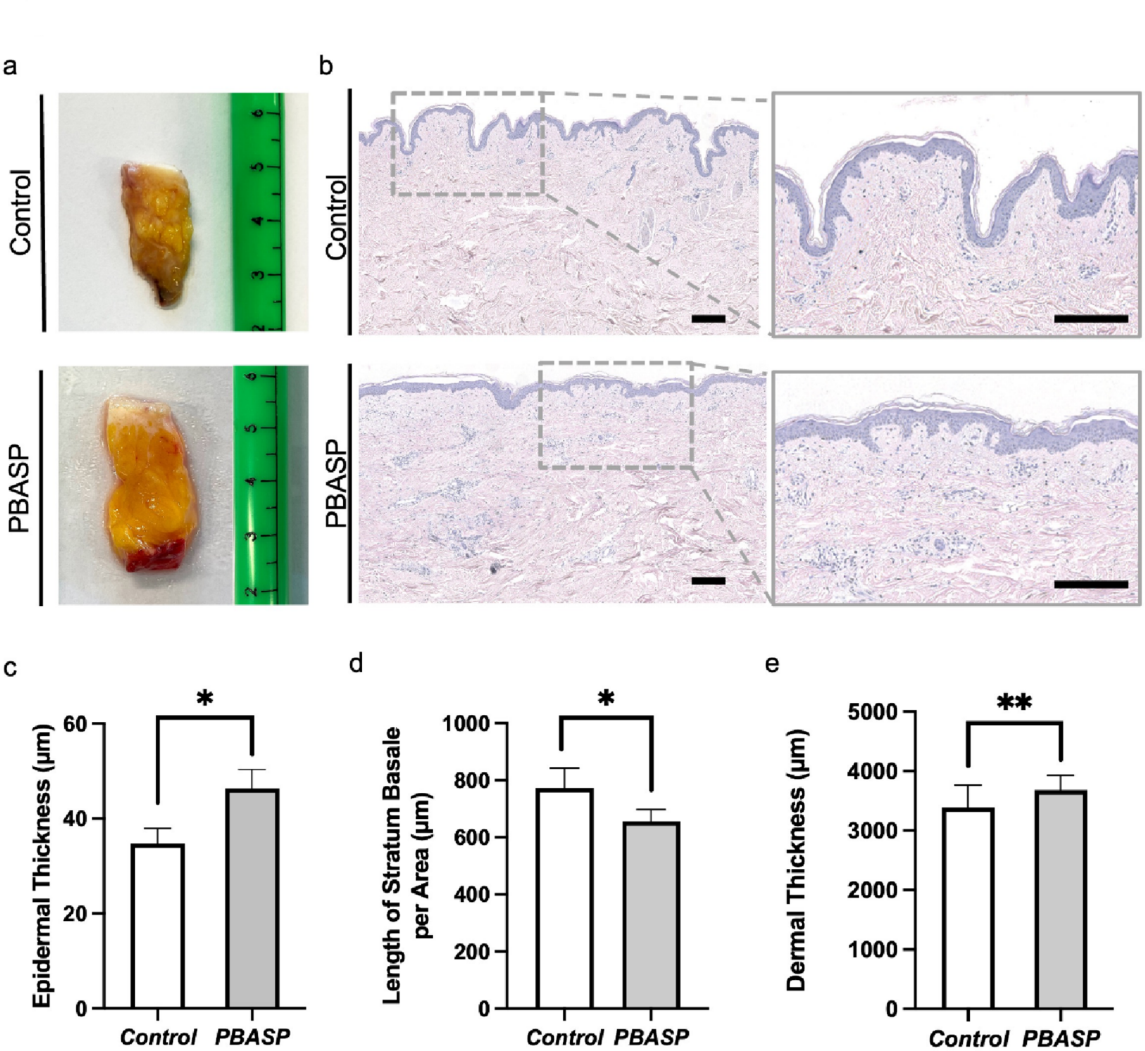
Comparison of healthy skin and PBASP tissue, including histological and morphological analysis. (**a**) Image comparing healthy skin with perforator-based adipocutaneous skin paddle (PBASP) tissue following harvesting, highlighting the swollen appearance and oily texture of the PBASP. (**b**) Hematoxylin and Eosin (H&E) staining of representative skin sections from control and PBASP tissue. Scale bars: 200 μm. (**c**) Quantification of epidermal thickness in control and PBASP tissue. (**d**) Measurement of basal membrane length per unit area in control and PBASP tissue. (**e**) Quantification of dermal thickness in control and PBASP tissue. Data are presented as mean ± SEM. *P < 0.05, **P < 0.01. *n* = 15 independent patient-derived paired samples (Day 0 control vs. PBASP). PBASP in this figure represents the pooled intervention cohort (postoperative Days 7–14).

Macroscopically, harvested PBASP tissues appeared swollen, enlarged, and oily, with fluid accumulation and oil droplets on postoperative day (POD) 7–14 (Fig. 2a). On microscopic evaluation of the H&E stained epidermis, dermis, and subcutaneous tissue layers (Fig. 2b), the epidermis in PBASP samples showed a 34% increase in thickness, measuring 46.4 ± 4.0 μm compared to 34.7 ± 3.2 μm in controls (*p* = 0.03; Fig. 2c). Additionally, the epidermis appeared less papillated, with the basal membrane length showing a 15% decrease in length per area (772 ± 71 μm vs. 656 ± 41 μm, *p* = 0.04, Fig. 2d). In the dermal layer, PBASP tissues exhibited morphological loosening, with a significant increase in thickness compared to controls (3332.9 ± 350 μm vs. 3797.5 ± 270 μm, *p* = 0.008; Fig. 2e).

### Early lymphatic remodeling-associated with upregulation of lymphatic markers

**Fig. 3:**
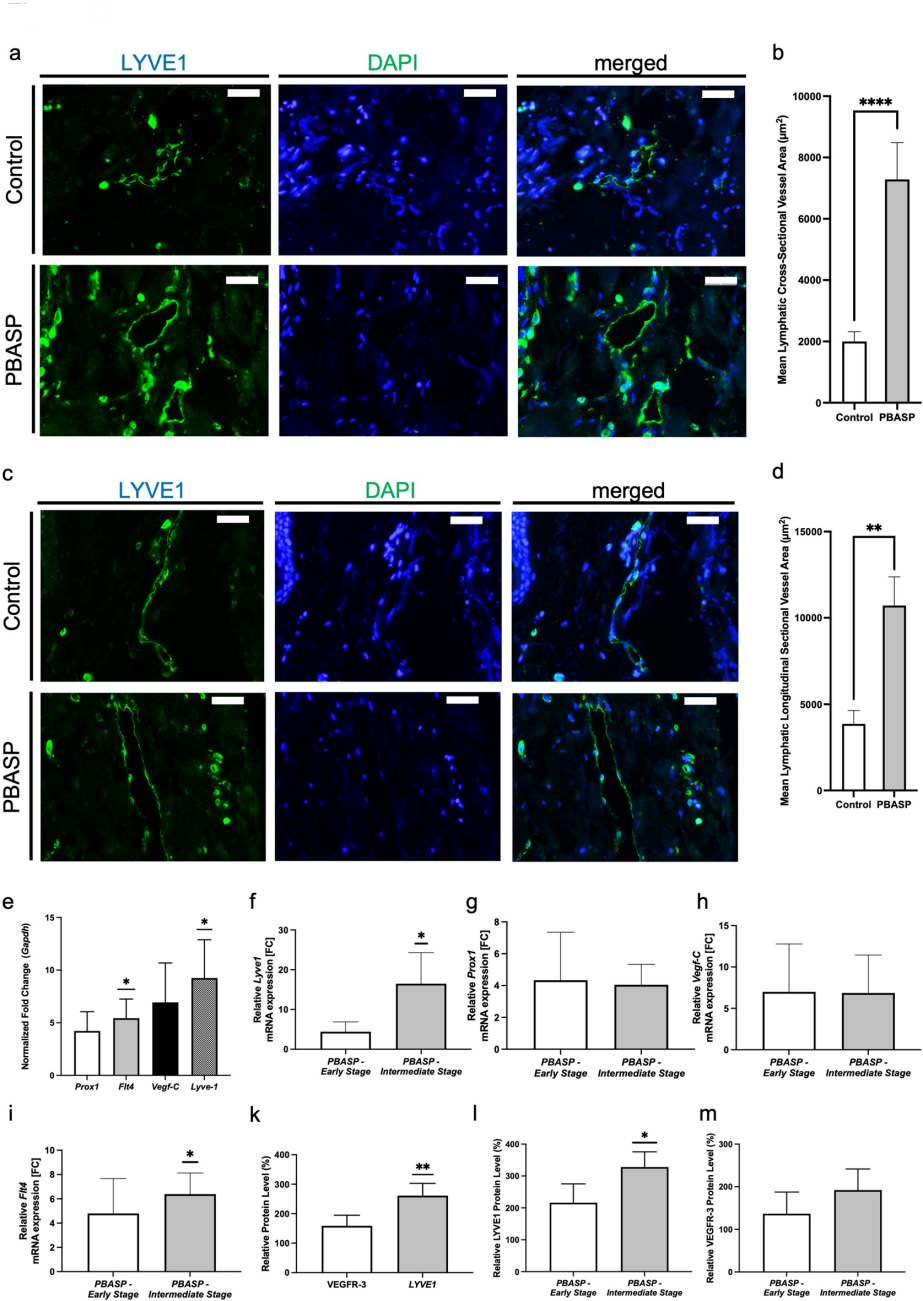
Lymphatic vessel analysis in control and PBASP samples. (**a**,**b**) Representative immunofluorescence images (A) and quantification (B) of mean lymphatic cross-sectional vessel area in control and perforator-based adipocutaneous skin paddle (PBASP) samples. Scale bars: 50 μm. Quantification was performed on 15 biological samples per group (Day 0, *n* = 15; PBASP, *n* = 15), with 1 analyzed section per sample and 4 high-power fields per section. Lymphatic vessels shown are located predominantly in the reticular dermis and near the dermo-hypodermal junction. (**c**,**d**) Representative immunofluorescence images (C) and quantification (D) of mean lymphatic longitudinal sectional vessel area in control and PBASP skin samples. Scale bars: 50 μm. Quantification was performed on 15 biological samples per group (Day 0, *n* = 15; PBASP, *n* = 15), with 1 analyzed section per sample and 4 high-power fields per section. Lymphatic vessels shown are located predominantly in the reticular dermis and near the dermo-hypodermal junction. (**e**) Relative mRNA expression of *Prox1*, *Flt4*, *Vegf-C*, and *Lyve1* in control and PBASP samples, measured by qPCR. Gene expression was normalized to *Gapdh* mRNA levels, with data shown relative to control. (**f**) Relative mRNA expression of *Lyve1* in early- and intermediate-stage PBASP subgroups compared to control, measured by qPCR. Gene expression was normalized to *Gapdh* mRNA levels. (**g**) Relative mRNA expression of *Flt4* in early- and intermediate-stage PBASP subgroups compared to control, measured by qPCR. Gene expression was normalized to *Gapdh* mRNA levels. (**h**) Relative mRNA expression of *Vegf-C* in early- and intermediate-stage PBASP subgroups compared to control, measured by qPCR. Gene expression was normalized to *Gapdh* mRNA levels. (**i**) Relative mRNA expression of *Prox1* in early- and intermediate-stage PBASP subgroups compared to control, measured by qPCR. Gene expression was normalized to *Gapdh* mRNA levels. (**k**) ELISA quantification of VEGFR-3 and LYVE1 protein levels in control and PBASP samples. Protein levels are shown relative to control. (**l**) Relative protein levels of LYVE1 in early- and intermediate-stage PBASP subgroups, with data shown relative to control. (**m**) Relative protein levels of VEGFR-3 in early- and intermediate-stage PBASP subgroups, with data shown relative to control. Data are presented as mean ± SEM. *P < 0.05, **P < 0.01, ****P < 0.0001. *n* = 15 independent patient-derived paired samples (Day 0 control vs. PBASP). Subgroups included *n* = 9 early-stage PBASP samples (Days 7–10) and *n* = 6 intermediate-stage PBASP samples (Days 11–14).

LYVE-1 staining revealed significant lymphatic vessel dilation in the PBASP tissues. LYVE-1 + vessels in the control samples appeared normal in morphology, while the PBASP tissues showed significant dilation and abnormal structures. The l LYVE-1 + vessels observed are primarily located in the reticular dermis and near the dermo-hypodermal junction. Morphometric analysis showed a 3.6-fold increase in LYVE-1 + vessel area in cross-Sect.  (1999 ± 317 μm^2^ vs. 7285 ± 1198 μm^2^, *p* < 0.0001; Fig. 3a,b). Longitudinal sections also showed a significant 2.7-fold increase in luminal area, from 3864 ± 771 μm^2^ to 10,714 ± 1659 μm^2^ (*p* = 0.001; Fig. 3c,d). At the molecular level, *Lyve-1* mRNA expression was significantly elevated in PBASP tissues, with a 9.3 ± 3.6-fold increase compared to controls (*p* = 0.02; Fig. 3e). Time-dependent analysis revealed a non-significant 4.4 ± 2.5-fold increase in the early-stage PBASP group (*p* = 0.36), which escalated to a significant 16.5 ± 7.8-fold increase in the intermediate-stage group (*p* = 0.03; Fig. 3f). To further substantiate lymphangiogenic activity, the expression of *Prox1*, *Flt4*, and *Vegf-C* was analyzed. *Flt4* expression rose significantly (5.4 ± 1.8-fold, *p* = 0.026; Fig. 3e), while *Prox1* and *Vegf-C* showed substantial trends toward increased expression, including in the subgroup analysis (Fig. 3e,g,h). Subgroup analysis of *Flt4* mRNA levels revealed a considerable 4.8 ± 2.9-fold increase in early-stage PBASP (*p* = 0.36), which rose significantly to 6.4 ± 1.7-fold in intermediate-stage PBASP (*p* = 0.03; Fig. 3i). Protein analysis confirmed a 261 ± 42% increase in LYVE1 protein synthesis across the experimental group (Fig. 3k). An increase of 216% ± 59% in early-stage samples was observed in the time-specific analysis, with a p-value of 0.05, and a significant increase in intermediate-stage PBASP (328 ± 48%, *p* = 0.03, Fig. 3l). VEGFR-3 protein levels showed non-significant increases in early- and intermediate-stage PBASP (Fig. 3m).

Because LYVE-1 can also be expressed by selected macrophage populations, and because LYVE-1/macrophage co-localization was not assessed, increased LYVE-1 mRNA and protein levels should be interpreted as lymphatic-associated signal changes rather than definitive evidence of increased lymphatic endothelial cell number or de novo lymphangiogenesis.

### Divergent endothelial marker changes without histological evidence of increased vessel density

To gain a comprehensive understanding of our model, we also investigated changes in angiogenesis within the PBASP. CD31 staining showed no significant difference in the proportion of endothelial cells, with the CD31-positive cells relative to the total number of DAPI-stained cells measuring 0.24 ± 0.03 in controls and 0.27 ± 0.03 in PBASP samples (*p* = 0.38; see Supplementary Fig. 1a,b, illustrating the results of the immunofluorescence evaluation). At the molecular level, Pecam1 relative mRNA expression was significantly elevated by 8.8 ± 3.5-fold compared with controls (*p* = 0.04), while *Flt1* expression increased 7.1 ± 2.2-fold but did not reach statistical significance (*p* = 0.05; see Supplementary Fig. 1c displaying the normalized mRNA levels of *Flt1* and *Pecam1*). Subgroup analysis revealed a significantly higher *Pecam*1 level in the intermediate-stage PBASP group, while *Flt1* expression showed no significant changes in either subgroup compared to controls (see Supplementary Fig. 1d,e). In the absence of a corresponding increase in CD31-defined endothelial cell density, these findings are interpreted cautiously as being more consistent with endothelial activation or phenotype/permeability-related shifts than with definitive angiogenesis.

### Dynamic inflammatory changes in PBASP

**Fig. 4:**
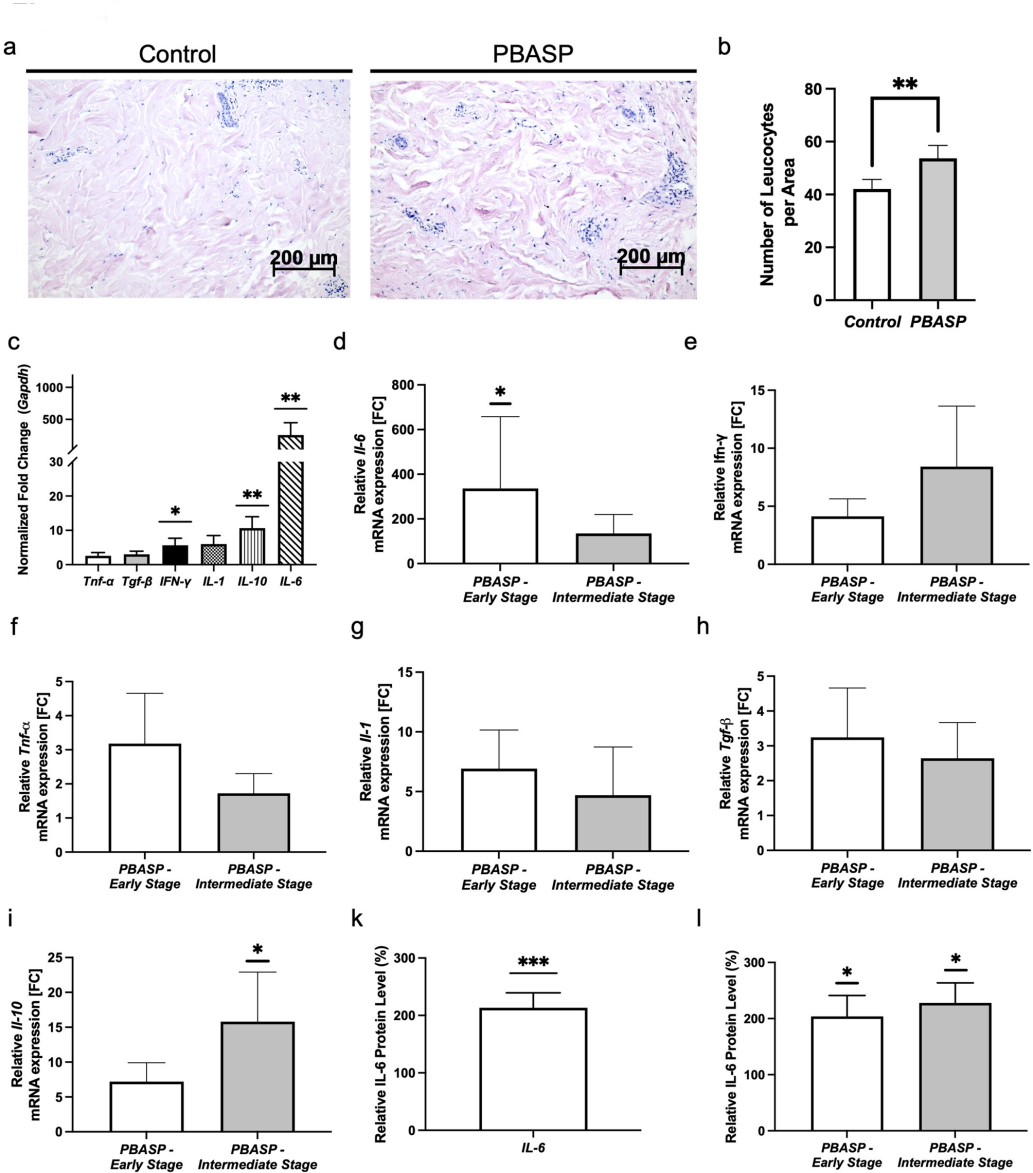
Leukocyte infiltration and inflammatory marker expression in control and PBASP tissues. (**a**) Hematoxylin and Eosin (H&E) staining of representative skin sections from control and perforator-based adipocutaneous skin paddle (PBASP) tissues, demonstrating leukocyte infiltration. Scale bars: 200 μm. (**b**) Quantification of leukocyte number per area in H&E-stained sections of control and PBASP tissues. (**c**) Relative mRNA expression of *Tnf-α*,* Tgf-β*,* Ifn-γ*,* Il-1*,* Il-10*, and *Il-6* in control and PBASP tissues, measured by qPCR. Gene expression was normalized to Gapdh, with data shown relative to control. (**d**) Relative mRNA expression of *Il-6* in early- and intermediate-stage PBASP subgroups compared to control, measured by qPCR and normalized to Gapdh expression. (**e**) Relative mRNA expression of Ifn-γ in early- and intermediate-stage PBASP subgroups compared to control, measured by qPCR and normalized to Gapdh expression. (**f**) Relative mRNA expression of Tnf-α in early- and intermediate-stage PBASP subgroups compared to control, measured by qPCR and normalized to Gapdh expression. (**g**) Relative mRNA expression of Il-1 in early- and intermediate-stage PBASP subgroups compared to control, measured by qPCR and normalized to Gapdh expression. (**h**) Relative mRNA expression of Tgf-β in early- and intermediate-stage PBASP subgroups compared to control, measured by qPCR and normalized to Gapdh expression. (**i**) Relative mRNA expression of *Il-10* in early- and intermediate-stage PBASP subgroups compared to control, measured by qPCR and normalized to Gapdh expression. (**j**) ELISA quantification of IL-6 protein levels in control and PBASP tissues, with protein levels shown relative to control. (**k**) Relative protein levels of IL-6 in early- and intermediate-stage PBASP subgroups, presented relative to control values. Data are presented as mean ± SEM. *P < 0.05, **P < 0.01, ***P < 0.001. *n* = 15 independent patient-derived paired samples (Day 0 control vs. PBASP). Subgroups included *n* = 9 early-stage PBASP samples (Days 7–10) and *n* = 6 intermediate-stage PBASP samples (Days 11–14).

Microscopic analysis revealed significantly higher leukocyte infiltration in the PBASP samples (Control: 42.1 ± 3.6 versus PBASP: 53.7 ± 4.9; *p* = 0.002; Fig. 4a,b). At the molecular level, *Il-6* mRNA expression increased significantly by 255.9 ± 192.6-fold (*p* = 0.002; Fig. 4c), with the highest increase observed in the early stage (336.5 ± 321.3-fold, *p* = 0.0195; Fig. 4d). *Ifn-γ* expression showed a significant overall increase (5.7 ± 2.1-fold, *p* = 0.0402; Fig. 4c), with a 4.1 ± 1.5-fold rise in the early stage (*p* = 0.19) and a considerable increase to 8.4 ± 5.2-fold in the intermediate stage (*p* = 0.15; Fig. 4e).

The inflammatory markers *Tnf-α* (2.6 ± 0.9-fold), *Il-1* (6.0 ± 25-fold), and *Tgf-β* (3.0 ± 0.9-fold) showed modest increases, but none reached statistical significance (Fig. 4c). Subgroup analyses also revealed no significant differences compared to controls (Fig. 4f–h).

As part of the anti-inflammatory response, *Il-10* showed a significant 10.7 ± 3.3-fold increase in expression (*p* = 0.008), indicating a compensatory response. Time-specific analysis revealed a consistent rise from 7.2 ± 2.7-fold in early-stage samples (*p* = 0.12) to 15.8 ± 7.1-fold in the intermediate stage (*p* = 0.037; Fig. 4i).

Protein analysis confirmed significant IL-6 upregulation (214 ± 26%, *p* = 0.0006; Fig. 4k). Subgroup analysis mirrored mRNA trends, with significant increases in the early stage (204 ± 37%, *p* = 0.027) and intermediate stage (228 ± 36%, *p* = 0.031; Fig. 4l).

### Fibrotic and adipogenic remodeling in PBASP tissue

**Fig. 5:**
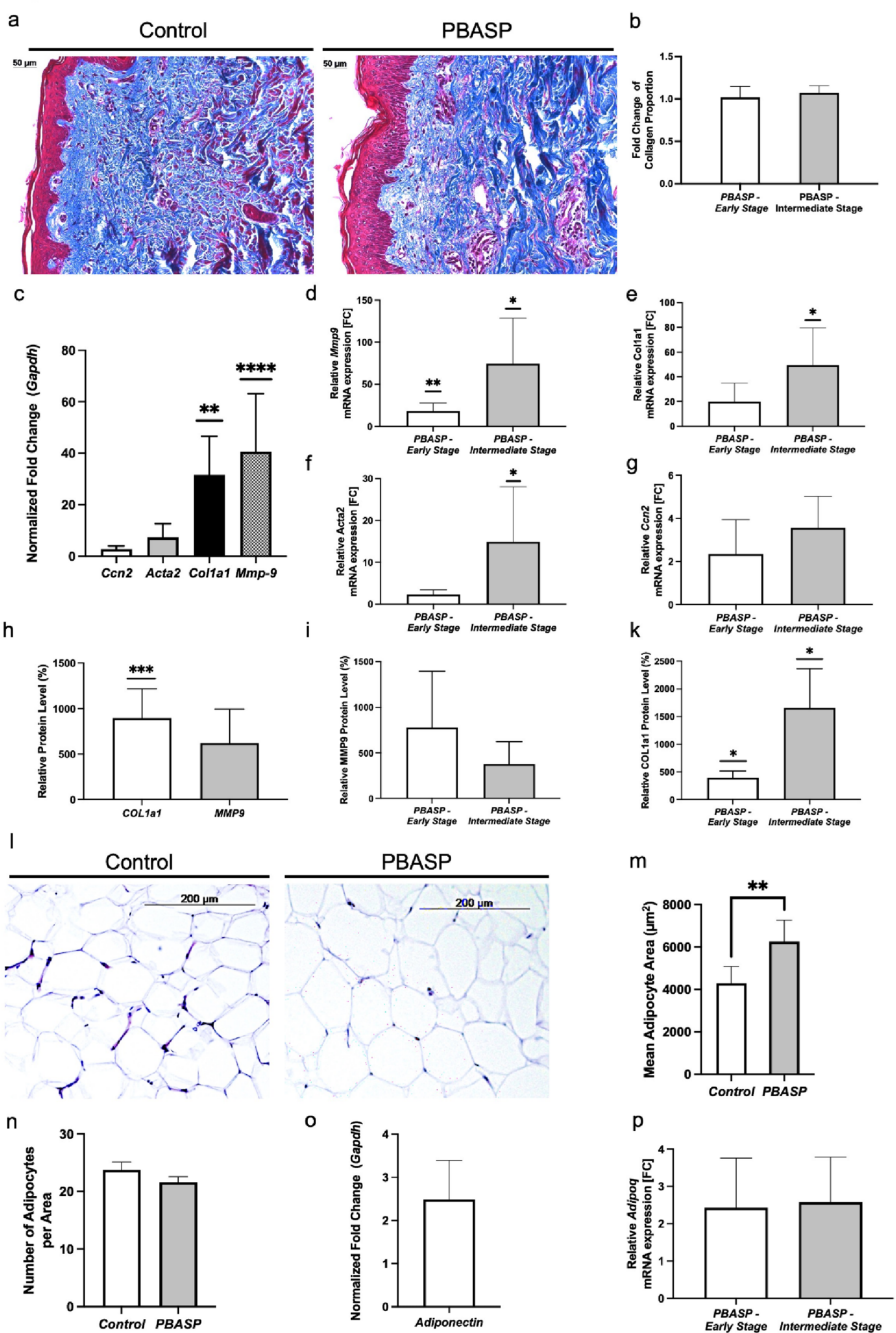
Fibrotic and adipocutaneous tissue remodeling in control and PBASP samples. (**a**) Masson’s trichrome staining of skin sections from control and perforator-based adipocutaneous skin paddle (PBASP) tissues. Scale bars: 50 μm. (**b**) Quantification of collagen proportion in control and PBASP tissues. (**c**) Relative mRNA expression of *Ccn2*, *Acta2*, *Col1a1*, and *Mmp-9* in control and PBASP tissues, measured by qPCR. Gene expression was normalized to Gapdh, with data shown relative to control. (**d**) Relative mRNA expression of *Mmp-9* in early- and intermediate-stage PBASP subgroups compared to control, measured by q PCR and normalized to Gapdh expression. (**e**) Relative mRNA expression of *Col1a1* in early- and intermediate-stage PBASP subgroups compared to control, measured by qPCR and normalized to Gapdh expression. (**f**) Relative mRNA expression of *Acta2* in early- and intermediate-stage PBASP subgroups compared to control, measured by qPCR and normalized to Gapdh expression. (**g**) Relative mRNA expression of *Ccn2* in early- and intermediate-stage PBASP subgroups compared to control, measured by qPCR and normalized to Gapdh expression. (**h**) ELISA quantification of COL1A1 and MMP9 protein levels in control and PBASP tissues, with protein levels shown relative to control. (**i**) Relative protein levels of MMP9 in early- and intermediate-stage PBASP subgroups, presented relative to control values. (**k**) Relative protein levels of COL1A1 in early- and intermediate-stage PBASP subgroups, presented relative to control values. (**l**) H&E staining of representative subcutaneous tissue sections from control and PBASP tissues. Scale bars: 200 μm. (**m**) Quantification of mean adipocyte area. (**n**) Quantification of adipocyte count per unit area. (**o**) Relative mRNA expression of *Adipoq* in control and PBASP tissues, measured by qPCR. Gene expression was normalized to Gapdh, with data shown relative to control. (**p**) Relative mRNA expression of Adipoq in early- and intermediate-stage PBASP subgroups compared to control, measured by qPCR and normalized to Gapdh expression. Data are presented as mean ± SEM. *P < 0.05, **P < 0.01, ***P < 0.001, ****P < 0.0001. *n* = 15 independent patient-derived paired samples (Day 0 control vs. PBASP). Subgroups included *n* = 9 early-stage PBASP samples (Days 7–10) and *n* = 6 intermediate-stage PBASP samples (Days 11–14).

Masson Trichrome staining revealed no significant differences in collagen proportion microscopically, though tissue thickening and loosening were observed (Fig. 5a,b). At the molecular level, fibrosis-associated markers showed significant upregulation. MMP-9, a key enzyme involved in ECM remodeling and collagen degradation, exhibited a 40.6 ± 22.5-fold increase in expression across all PBASP samples (*p* < 0.0001; Fig. 5c). Subgroup analysis revealed a progression of *Mmp-9* expression, with an 18.2 ± 9.4-fold increase in the early-stage group (*p* = 0.004) that escalated to 74.3 ± 54.4-fold in the intermediate-stage group (*p* = 0.03; Fig. 5d). Similarly, *Col1a1*, a primary component of the ECM, was significantly upregulated in PBASP tissues, with a 31.6 ± 14.9-fold increase in expression (*p* = 0.005; Fig. 5c). *Col1a1* expression was reduced during the early stage (19.9 ± 15.0-fold, *p* = 0.30), but significantly increased during the intermediate stage (49.3 ± 30.3-fold, *p* = 0.03; Fig. 5e). A similar pattern was observed for the myofibroblast marker *Acta2* (9.56 ± 7.89-fold, *p* = 0.03; Fig. 5f). *Ccn2* showed no significant changes across the experimental group or subgroups (Fig. 5g). These molecular changes are consistent with early ECM activation within the Days 7–14 observation window, but they do not establish sustained remodeling or irreversible fibrosis.

Protein-level analyses supported these findings. MMP-9 protein levels increased by 349 ± 136%, although the changes were not statistically significant (*p* = 0.33; Fig. 5h). MMP-9 protein levels were elevated in the early-stage group (329 ± 168%, *p* = 0.55) before slightly descending in the intermediate stage (379 ± 246%, *p* = 0.44; Fig. 5i). In contrast, COL1A1 protein levels were significantly elevated across the entire PBASP group, with a 669 ± 141% increase (*p* = 0.0003; Fig. 5h). Subgroup analysis showed a steady rise in COL1A1 protein expression, increasing from 393 ± 124% in the early stage (*p* = 0.02) to 1068 ± 218% in the intermediate stage (*p* = 0.03; Fig. 5k). Because follow-up ended at postoperative Days 7–14, these data should be interpreted as early remodeling-associated changes rather than evidence of sustained long-term fibrosis.

Alongside fibrous remodeling, PBASP tissues also exhibited adipogenic changes. Adipocyte size increased significantly, with median values rising from 4,283 ± 796 μm² in controls to 6,248 ± 1,017 μm^2^ in PBASP tissues, representing a 46% increase (*p* = 0.002; Fig. 5l,m). Adipocyte density showed a slight reduction in PBASP samples (24 ± 1% vs. 22 ± 1%, *p* = 0.06; Fig. 5n).

Adipogenic marker adiponectin (Adipoq) showed no significant upregulation across PBASP samples on mRNA level (2.5 ± 0.9-fold, *p* = 0.80, Fig. 5o). Subgroup analysis similarly revealed no significant differences between early- and intermediate-stage groups (Fig. 5p).

## Discussion

The molecular pathophysiology and progression of secondary lymphedema remain incompletely understood, and human tissue-based research is limited by restricted access to temporally defined samples. In this context, the PBASP model provides a PBASP-derived, lymph-outflow–deficient human tissue platform that captures early pathological features of secondary lymphedema. Its key novelty lies in the ability to perform prospective intraindividual paired sampling at defined short intervals after lymphatic interruption (Day 0 vs. postoperative Days 7–14) in viable human tissue with preserved arterial and venous perfusion. Using histological, immunofluorescence, transcript-level, and protein-level analyses, we found that PBASP tissues develop edema, epidermal and dermal thickening, enlarged LYVE-1 + vessel profiles, inflammatory activation, and early remodeling changes. These findings are compatible with early localized lymphatic insufficiency, but they do not prove functional lymphatic impairment and should not be interpreted as equivalence to established chronic human lymphedematous tissue^20,21^.

Lymphedema is defined by impaired lymph transport and may already exist in an early stage before overt chronic fibroadipose remodeling is fully established^22,23^. By contrast, ordinary postoperative swelling reflects the expected acute wound-healing response, driven predominantly by sterile inflammation, transient capillary leakage, and endothelial permeability changes, with subsequent resolution as healing progresses^24^. Importantly, postoperative inflammatory mediators such as IL-6 are not specific for lymphedema, because IL-6 is a well-established marker of surgical tissue injury and rises in proportion to operative trauma. We therefore do not interpret the swollen macroscopic appearance, IL-6 increase, leukocyte infiltration, or matrix-marker induction in isolation as proof of lymphedema. Rather, PBASP should be interpreted cautiously as a local, hypothesis-generating model in which a tissue island has preserved arterial and venous perfusion but lacks physiological lymphatic reconnection. Because direct functional lymphatic drainage was not measured and no maintained-lymphatic-outflow control island was included, the present data cannot definitively distinguish early lymphatic insufficiency from sterile postoperative edema or wound-repair-associated inflammation.

This cautious interpretation is also supported by the combined histological and molecular pattern but is limited by the nonspecificity of individual findings. Typical postoperative swelling is characterized by interstitial edema, vascular congestion, and a temporally resolving inflammatory infiltrate within the broader program of re-epithelialization, granulation tissue formation, angiogenesis, and collagen deposition^25,26^. Lymphedema arises from impaired clearance of protein-rich interstitial fluid and is accompanied by lymphatic vessel dilation, skin thickening, inflammatory persistence, and progressive matrix and adipose remodeling^27,28^. The PBASP phenotype showed several changes compatible with the latter process, including epidermal and dermal thickening, a 3.6-fold increase in LYVE-1 + lymphatic vessel area, increased *Lyve1* and *Flt4* expression, upward trends in Prox1 and *Vegf-C*, and early induction of *Mmp9*,* Col1a1*,* Acta2*, and adipocyte hypertrophy. However, these findings can also overlap with sterile inflammation, wound repair, and vascular edema. Therefore, PBASP is best described as a hypothesis-generating model of early localized lymphatic insufficiency.

We acknowledge that wound healing itself can activate a VEGF-C/VEGFR3-dependent lymphangiogenic program^29,30^. However, in normal repair this response supports restoration of drainage and resolution of inflammatory edema^29^. In PBASP, lymphatic-associated signaling emerged in tissue that was not physiologically reconnected to lymphatic outflow and was accompanied by enlarged LYVE-1 + vessel profiles and early remodeling-associated changes over postoperative Days 7–14. Nevertheless, because lymphatic drainage was not directly measured, these findings should be interpreted as lymphatic-associated remodeling in a presumed no-outflow context, not as direct proof of lymphatic stasis or functional lymphatic failure.

Early lymphatic remodeling was a defining feature of the PBASP model. Morphological evidence of enlarged LYVE-1 + vessel profiles, supported by a 3.6-fold increase in LYVE-1 + cross-sectional vessel area and elevated expression of *Vegf-C* and *Flt4*, indicates an early lymphatic-marker response. However, as lymphatic vessel density, endothelial proliferation, lymphatic drainage function, and LYVE-1 co-localization with macrophage markers were not assessed, these findings should not be interpreted as direct evidence of de novo lymphangiogenesis or increased lymphatic endothelial cell number. These results are compatible with reports from patient biopsies showing altered lymphatic morphology and congestion^31^, but they remain hypothesis-generating in the absence of direct functional validation.

Angiogenesis in the PBASP model was limited. Endothelial changes in PBASP should be interpreted with caution. Although Pecam1 expression was significantly increased and *Flt1* showed a borderline rise at the transcript level, CD31 staining did not demonstrate a significant increase in endothelial cell density. This divergence argues against definitive neovascularization within the observed time window and is more consistent with endothelial activation and/or altered vascular permeability than with frank angiogenesis. Because alternative endothelial markers, perivascular coverage analyses, and automated morphometry were not included in the present study, the vascular component of the PBASP response should be regarded as preliminary and hypothesis-generating. Future studies should incorporate expanded endothelial phenotyping to better distinguish endothelial activation from true angiogenic remodeling.

Lymphatic stasis in lymphedema is associated with upregulation of inflammatory responses^32^. PBASP tissues exhibited significant leukocyte infiltration and elevated IFN-γ and IL-6 levels, particularly in early stages, paralleling clinical findings in patients^33,34^. IL-6 contributes to adipose deposition and chronic inflammation, while IFN-γ impairs lymphatic regeneration^35^. However, IL-6 is also a recognized marker of postoperative tissue injury. Therefore, these inflammatory changes should not be considered lymphedema-specific in isolation. Instead, they gain discriminatory value when interpreted in conjunction with the defined lymphatic outflow deficit, lymphatic vessel dilation, and the concurrent early remodeling phenotype observed in PBASP^24,27,28^.

Interestingly, IL-10 expression increased in later PBASP samples, suggesting a compensatory anti-inflammatory response. These temporal dynamics of cytokine expression, which align with human lymphedema, are difficult to assess in patient tissue due to sampling limitations. Rodent studies often rely on homogeneous genetic backgrounds and forced immune modulation, while PBASP naturally reflects human immune variability and allows observation of innate immune dynamics^36^.

Chronic inflammation in lymphedema initiates tissue remodeling, resulting in progressive fibrosis characterized by excessive collagen deposition and the expansion of adipose and connective tissues^37^. In PBASP, early remodeling-associated changes were characterized by upregulation of ECM-remodeling and collagen-associated markers, including MMP-9 and COL1A1. While histological collagen deposition remained unchanged within the observation period, these gene-level changes are consistent with early ECM activation. Such findings are consistent with the known progression of lymphedema from inflammation to fibrosis, where MMP activity and type I collagen synthesis precede visible tissue stiffening^38^. However, because the present observation period was limited to postoperative Days 7–14, the data do not determine whether these changes would persist, resolve, or progress to established fibrosis. Accordingly, statements regarding sustained remodeling or lack of spontaneous recovery have been removed.

Adipogenic remodeling in PBASP was characterized by significant adipocyte hypertrophy without a decrease in cell number, indicating early adipose expansion. Expression of adiponectin, though not statistically significant, suggested emerging metabolic activation. These observations parallel early adipose changes seen in human lymphedema biopsies and imaging studies^39^, but they are not specific for lymphedema and may also reflect postoperative inflammatory and metabolic responses. Clinical research shows that stagnant lymph, rich in lipids and cytokines, promotes adipogenesis. IL-6, elevated in PBASP, is also linked to increased adipose deposition^40^. However, these findings align with evidence that adipogenic responses typically manifest weeks after injury, which explains their limited detectability during the early and intermediate stages of our model^41^.

The PBASP model addresses multiple limitations of current research approaches. Unlike clinical studies, which are restricted by ethical considerations and inter-patient variability, PBASP allows intraindividual, longitudinal comparisons with clear time points post-lymphatic injury. Compared to animal models, it avoids species-specific anatomical and immunological discrepancies. However, because PBASP is removed within postoperative Days 7–14, this study cannot assess long-term spontaneous resolution, persistence, or progression. Each PBASP sample serves as a paired intraindividual comparator to Day 0 skin, minimizing some confounders and enabling focused investigation of early tissue responses associated with presumed local lymphatic outflow insufficiency^42^.

Prior human studies have provided important insights into lymphedematous tissue, including histopathological analyses of excised chronic secondary lymphedema tissue, paired biopsies of affected and unaffected tissue obtained during lymphatic reconstructive procedures, and molecular or multi-omics analyses of chronic lymphedematous tissue after cancer treatment^43–46^. However, these approaches generally assess established disease and do not provide a prospective, short-interval, intraindividual model immediately following a defined lymphatic outflow interruption. By contrast, PBASP enables paired sampling of viable human tissue in the early post-interruption phase, thereby complementing prior human surgical contexts rather than replacing them.

Clinically, PBASP is easily implemented. It requires no additional surgery, causes no harm, and can be removed at bedside. This makes it ethically viable and suitable for routine collection. Despite its strengths, the model does not capture the chronic features of secondary lymphedema (sLE), such as advanced fibrosis and adipose tissue accumulation, owing to the limited observational period. Accordingly, PBASP should be interpreted as a hypothesis-generating model of early localized lymphatic insufficiency in a PBASP-specific reconstructive context. The findings should not be directly extrapolated to established chronic lymphedema, all etiologies of secondary lymphedema, or all reconstructive settings. Additionally, the relatively small sample size—constrained by clinical availability—reduces the statistical power for detailed subgroup analyses.

A conceptual limitation of the PBASP model is that it does not fully reproduce the anatomical pattern of typical cancer-related secondary lymphedema. In most clinical settings, lymphedema follows a proximal interruption of lymphatic drainage after lymph node dissection and/or radiotherapy, with variable distal rerouting or collateralization that can influence disease severity^47–49^. By contrast, PBASP represents a lymphatically isolated local tissue compartment with preserved arterial and venous perfusion but no physiological lymphatic outflow. This confined setting may generate local edema and lymphatic-associated structural changes, but it does not fully capture whole-limb phenomena such as collateral drainage, hydrostatic load, muscle-pump effects, or late chronic remodeling. We have therefore avoided attributing LYVE-1 + vessel enlargement to increased endolymphatic pressure, because lymphatic inflow, contractility, and pressure were not measured in PBASP. Accordingly, PBASP should be interpreted as a local hypothesis-generating platform for early human tissue responses after presumed lymphatic outflow insufficiency, with limited generalizability to established limb lymphedema after proximal lymphatic injury.

Systemic inflammation and patient-level comorbidities may have influenced cytokine expression and lymphangiogenic signaling. Relevant clinical variables, including trauma, infection, obesity, diabetes, smoking status, and history of malignancy, were recorded and are summarized in Supplementary Table 2. However, because of the limited cohort size, these factors could not be controlled for in adjusted or subgroup analyses. Although the paired intraindividual design reduces interindividual confounding, residual effects of systemic inflammatory status and comorbidities cannot be excluded.

A major limitation is the absence of direct functional validation of lymphatic impairment. Near-infrared fluorescence indocyanine green lymphangiography, ex vivo tracer-clearance assays, lymphoscintigraphy, or anatomical mapping of isolated lymphatic drainage pathways were not performed. Therefore, although the surgical configuration suggests absence of physiological lymphatic reconnection and the histological and molecular findings are compatible with early lymphatic insufficiency, the study does not directly demonstrate absent lymphatic drainage or distinguish PBASP definitively from post-surgical edema. In addition, the study lacks an experimental control in which lymphatic outflow is maintained. These limitations should be addressed in future prospective studies before PBASP can be considered a validated model of human lymphedema.

Looking forward, PBASP opens new avenues for translational applications. Ex vivo perfusion of PBASP tissue post-removal could enable drug testing in viable lymphedematous human tissue. Topical or injected therapies targeting IL-6, IL-10, or TGF-β pathways could be evaluated for their effect on inflammation, fibrosis, or lymphangiogenesis. Moreover, PBASP could be used to study the impact of early physical therapy interventions like manual lymph drainage on cellular and molecular outcomes^50^. As a controllable and human-relevant model, PBASP may also support imaging validation, biomarker discovery, and testing of advanced therapies such as gene or cell-based regenerative approaches.

## Conclusion

The PBASP model represents a PBASP-derived human tissue platform for studying early tissue responses after presumed localized lymphatic outflow insufficiency. Its main strengths are preserved arterial and venous perfusion, absence of physiological lymphatic reconnection by surgical design, and the ability to perform short-interval intraindividual paired sampling with multimodal tissue profiling. The model captures structural, lymphatic-marker, inflammatory, and early remodeling-associated changes that are compatible with early lymphatic insufficiency. However, because direct functional lymphatic drainage was not measured and chronic disease features are not captured, PBASP should be considered a hypothesis-generating translational platform rather than a validated equivalent of established human lymphedematous tissue.

## Materials and methods

### Ethics declaration

This study was performed in accordance with the Declaration of Helsinki. Collection of human tissue samples for this study was approved as part of the study protocol. This human study was approved by Rhineland-Palatinate ethics committee-approval: approval no. 2020 15,173. All adult participants provided written informed consent to participate in this study. Written informed consent for the use of patient images has also been obtained and is archived.

### Sample collection

Tissue samples were collected from all consecutive patients undergoing free microvascular muscle flap procedures with PBASP at the Department of Hand, Plastic, and Reconstructive Surgery, BG Klinik Ludwigshafen, Germany, over a 12-month period. Intraoperative skin samples were taken from excess tissue, with follow-up samples obtained 7–14 days later during bedside PBASP removal (Fig. 1f). Only PBASPs with consistently normal perfusion and no clinical complications were included (Fig. 1g). Inclusion criteria were age > 18 years, ASA score < 5, no major burns (> 20%), no local skin lesions, and no active infection or sepsis. No objective measurements of tissue volume or weight were performed. This was because sample sizes were not comparable across time points: Day 0 samples were consistently smaller, as they were obtained from excess tissue during flap harvest, whereas PBASP samples collected at follow-up were variable in size. Accordingly, macroscopic changes were assessed qualitatively.

### Histological staining

Tissue samples were fixed in 4% paraformaldehyde (hazard: toxic; handled in fume hood with appropriate PPE), were dehydrated, and paraffin-embedded using HistoCore Arcadia H (Leica Biosystems, Germany). Section (5 μm) were stained with hematoxylin and eosin (H&E) or Masson Trichrome using standard protocols^51^. ImageJ software (v1.54p, NIH, USA) was used to measure epidermal and dermal thickness and basal membrane length. Stained slides were imaged in their entirety by microscopy. The region of interest containing the full epidermis and dermis was divided into three sections (left, center, and right). Thickness was measured in each section, with the left and right measurements taken at the respective image borders and the central measurement taken in the middle of the image. The mean of the three measurements was used for further analysis. Subsequently, differences between paired Day 0 and PBASP samples were calculated. Leukocytes and adipocytes were quantified from H&E-stained sections, and collagen content was assessed from Masson’s Trichrome-stained sections using ImageJ based on a validated protocol^52^.

### Immunofluorescence staining

Antigen retrieval was performed on tissue sections (5 μm), followed by blocking with 3% BSA in PBS. Sections were incubated overnight with primary antibodies LYVE-1 (1:200, CSB-PA897574EA01HU, polyclonal rabbit IgG, Cusabio, USA) and CD31/PECAM1 (1:50, AF3625, polyclonal goat IgG, R&D Systems, USA). Corresponding secondary antibodies—Goat Anti-Rabbit IgG H&L Alexa Fluor^®^ 488 (1:500, ab150077, Abcam, UK) and Donkey Anti-Goat IgG H&L Alexa Fluor^®^ 568 (1:200, ab175474, Abcam, UK)—were applied accordingly. Nuclei were stained with Vectashield Antifade Mounting Medium with DAPI (Vector Laboratories, USA). Negative controls consisted of sections processed identically but with omission of the primary antibodies, while the corresponding secondary antibodies were applied under the same conditions. No specific fluorescence signal was observed in these controls. Fluorescent images were acquired with a Zeiss Axio Imager M2 microscope (Zeiss, Germany). LYVE-1-positive vessel size and CD31-positive cells were quantified using ImageJ, following established protocols^52^. For immunofluorescence quantification, 15 biological samples per group were analyzed (Day 0, *n* = 15; PBASP, *n* = 15). One section per sample and four high-power fields per section were evaluated. Prior to assessment, the high-power fields were de-identified by an independent investigator and analyzed in random order. After image assessment, the results were re-identified and reassigned to the corresponding sample groups for quantification. Lymphatic vessels were identified predominantly in the reticular dermis and toward the dermo-hypodermal junction. No separate quantification of vessel density or caliber by dermal layer was performed. Absolute vessel depth was not measured because variable flap composition and skin thickness precluded standardized comparison across samples.

### qPCR

RNA was isolated using the RNeasy Fibrous Tissue Mini Kit (Qiagen, Germany), and cDNA was synthesized with the Maxima H Minus First Strand cDNA Synthesis Kit (Thermo Fisher Scientific). qPCR was performed with SYBR Green I Master Mix (Thermo Fisher Scientific, USA) on a LightCycler 480 II system (Roche, Switzerland). GAPDH served as the housekeeping gene. Primer sequences for qPCR are provided in Supplementary Table 1. Fold changes in gene expression were calculated using the ∆∆CT method and analyzed with GraphPad Prism 8 (GraphPad Software Inc., USA).

### Protein isolation and quantification

Frozen tissue samples were homogenized in RIPA buffer (hazard: irritant; handled with PPE) with protease inhibitors (Thermo Fisher Scientific, USA). Protein concentrations were measured using the Pierce BCA Protein Assay Kit (Thermo Fisher Scientific, USA). LYVE-1 (ELH-LYVE1, RayBiotech, USA), IL-6 (ELH-IL6-CL, RayBiotech, USA), MMP9 (SEA553Hu, CloudClone, USA), COL1A1 (SEA350Hu, CloudClone, USA) and VEGFR-3 (SEB893Hu, CloudClone, USA) were quantified using ELISA kits, following the manufacturers’ protocols. Assays were performed in duplicate, with standards and negative controls, and absorbance was read at 450 nm (Glomax Discover, Promega, USA).

### Statistical analysis

Sample-related data were organized by flap type, age group, BMI, and POD on which the PBASP was removed. In total, 15 patients were included, providing 15 interventional PBASP samples and 15 intraindividual, non-interventional skin samples (Day 0), which served as paired controls. For subgroup analyses, 9 PBASP samples were categorized as early-stage (Days 7–10) and 6 as intermediate-stage (Days 11–14).

All values are presented as mean ± SEM unless otherwise noted. Normality of distributions was assessed using the Shapiro–Wilk test. For normally distributed data, parametric tests were applied; when normality assumptions were not met, non-parametric alternatives were used, including the Wilcoxon test for paired comparisons, the Mann–Whitney U test for independent groups, and the Kruskal–Wallis test for multiple groups. Where multiple comparisons were performed, post hoc analyses with Dunn’s multiple comparison test were applied to control the type I error rate. All tests were two-tailed, with statistical significance defined as *p* < 0.05, and exact *p* values are reported. Winsorization was applied to adjust outliers to the 5th and 95th percentiles. Statistical analyses were conducted using GraphPad Prism 8 (GraphPad Software Inc., USA). For immunofluorescence quantification, 15 biological samples per group were analyzed (Day 0, *n* = 15; Day 7, *n* = 15). One section per sample and four high-power fields per section were evaluated.

## Supplementary Information

Below is the link to the electronic supplementary material.

Supplementary Material 1

## Data Availability

All data supporting the conclusions of this manuscript are provided in the figures and supplementary materials. No large-scale datasets were generated or analyzed. Additional datasets used or analyzed during the current study are available from the corresponding author upon reasonable request.
