# Supplementary material for: A perforator-based adipocutaneous skin paddle derived from free muscle flap reconstruction as a hypothesis-generating human tissue model of early localized lymphatic insufficiency
Source: Sci Rep. 2026 Aug 5;16:24200. doi: 10.1038/s41598-026-64775-3 (PMC13444241; doi:10.1038/s41598-026-64775-3)
Supplement: Supplementary file 1 [file 41598_2026_64775_MOESM1_ESM.pdf]

Supplementary Table 1

Used Primer Sequences

Gene	Primer sequence forward	Primer sequence reverse
Acta2	5’- CTgTTCCAgCCATCCTTCAT	5’- TCTTCCTgCCTgCCTTCTAA
Adipoq	5’- gggATTTCACCATgTTgTCC	5’- CTTCCCCTTCgCTCTTTCTT
Ccn2	5’- CCTggTCCAgACCACAgAgT	5’- TggAgATTTTgggAgTACgg
Col1a1	5’- gAgAgCATgACCgATggATT	5’- CCTTCTTgAggTTgCCAgTC
Flt1	5’-AgTTTAAAAggCACCCAgCA	5’-TCAgCCTTTCgCTTTCCTTA
Flt4	5’- AAgCTggTgATCCAgAATgC	5’- ACTTgTAgCTgTCggCTTgg
GAPDH	5’- CTCACTgTTCTCTCCCTCCg	5’- CTAACggCTgCCCATTCATT
Ifn-γ	5’- TgACCAgAgCATCCAAAAgA	5’- TTTCgCTTCCCTgTTTTAgC
IL-1a	5’- gCgTTTgAgTCAgCAAAgAAgT	5’- CATggAgTgggCCATAgCTT
IL10	5’- CCAgACATCAAggCgCATgT	5’- CATTCTTCACCTgCTCCACgg
Il6	5’- TACCCCCAggAgAAgATTCC	5’- TTTTCTgCCAgTgCCTCTTT
Kdr	5’-AAgTCgggAgAggAgAgAgg	5’-ggTgTTTTgCTgTgggAAAT
Lyve1	5’- CTACTCCTCCTgCTCCAgCTT	5’- ACCCAgCAgCTTCATTCTTg
Mmp9	5’- gACAAgCTCTTCggCTTCTg	5’- TgTCgCTgTCAAAgTTCgAg
Pecam1	5’-AgTCggACAgTgggACgTAT	5’-ACggACTTCgATggTCTgTC
Prx1	5’- TgTCATCTCACCACCTgAgC	5’- gATTgggTgACAATCCTTCC
Tgf-ß	5’- CCATCTAgCAAggTggCTTC	5’- gCggAAgTCAgAgAgTgAgg
Tnf- α	5’- gAggTgCTgACCgACAAAg	5’- ggAgggTgATggTgTCATCT
Vegf-C	5’-AAAgAACCTgCCCCAgAAAT	5’-gAAAATCCTggCTCACAAgC

List of forward and reverse primer sequences used for quantitative polymerase chain reaction (PCR) analysis of target genes: GAPDH, glyceraldehyde-3-phosphate dehydrogenase; IL, interleukin; TGF-β, transforming growth factor beta; TNF-α, tumor necrosis factor alpha; VEGF-C, vascular endothelial growth factor C; MMP-9, matrix metalloproteinase 9.

Supplementary Table 2

Patient comorbidities

**Comorbidities**	**n (%)**
Trauma	6 (40)
Infection	3 (20)
Obesity	6 (40)
Diabetes	4 (26.7)
Smoking	4 (26.7)
History of malignancy	6 (40)

Baseline patient comorbidities. Data are presented as n (%).

Supplementary Fig. 1

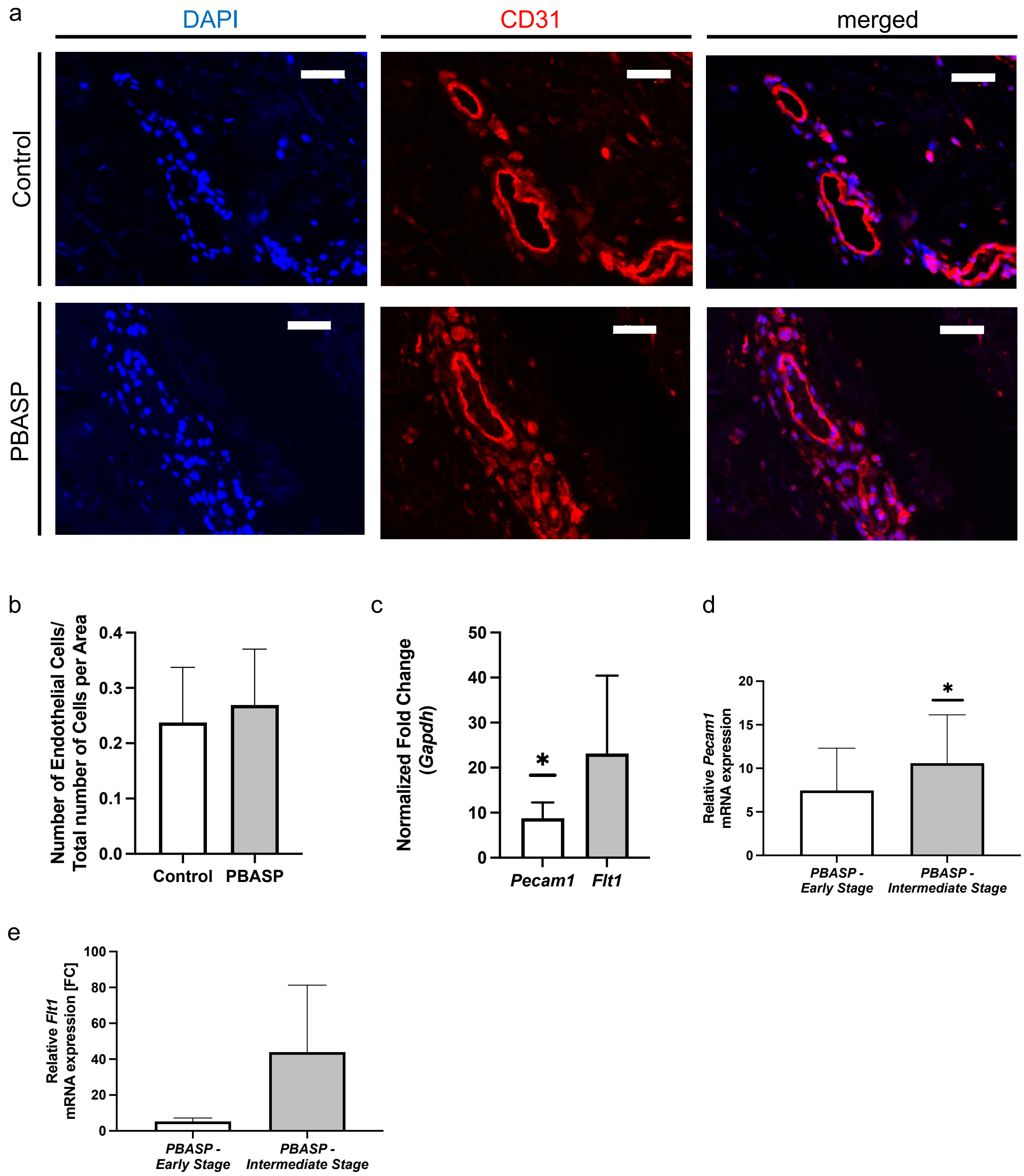

<u>Supplementary Figure 1</u>

Endothelial cell analysis in control and PBASP samples.

(a-b) Representative immunofluorescence images (A) and quantification (B) of endothelial cell number relative to total cells in control and perforator-based adipocutaneous skin paddle (PBASP) samples. Scale bars: 50 µm.

(c) Relative mRNA expression of *Pecam*1 and *Flt1* in control and PBASP samples, measured by qPCR. Gene expression was normalized to *Gapdh* mRNA levels, with data shown relative to control.

(d) Relative mRNA expression of *Pecam1* in early- and intermediate-stage PBASP subgroups compared to control, measured by qPCR. Gene expression was normalized to *Gapdh* mRNA levels.

(E) Relative mRNA expression of *Flt1* in early- and intermediate-stage PBASP subgroups compared to control, measured by qPCR. Gene expression was normalized to *Gapdh* mRNA *levels.*

*Data are presented as mean ± SEM. *P < 0.05. n = 15 independent patient-derived paired samples (Day 0 control vs. PBASP). Subgroups included n = 9 early-stage PBASP samples (Days 7–10) and n = 6 intermediate-stage PBASP samples (Days 11–14).*
